# Mendelian Randomization Analysis of Psoriasis and Multiparameter Renal Function Trajectories

**DOI:** 10.2174/0118715303426134251202143308

**Published:** 2026-05-21

**Authors:** Zhihao Huang, Shengxiu Liu

**Affiliations:** 1 Department of Dermatology, First Affiliated Hospital of Anhui Medical University, Hefei, Anhui, 230022, China

**Keywords:** Psoriasis, renal function, Mendelian randomization, stages G3 to G5, chronic kidney impairment, estimated glomerular filtration efficiency, dialysis

## Abstract

**Introduction:**

While observational studies suggest that psoriasis may increase the risk of renal impairment, evidence supporting a causal relationship remains inconclusive. This Mendelian randomization (MR) study systematically investigates the genetic causality between psoriasis and multidimensional renal function trajectories, encompassing both static measures and dynamic progression patterns.

**Methods:**

We utilized genetic variants associated with psoriasis identified in a Finnish population-based genome-wide study of 373,338 participants. Using a two-sample MR framework, we examined potential causal relationships across six kidney-related outcomes: (1) estimated glomerular filtration rate (eGFR), (2) albuminuria, (3) chronic kidney disease stages G3–G5 (CKD G3–G5), (4) need for dialysis, (5) accelerated progression to CKD (CKDi25), and (6) rapid deterioration in renal function (Rapid3). To ensure robustness, we applied MR-PRESSO for outlier correction and performed inverse-variance weighted (IVW) analyses under a random-effects model as the primary method, complemented by comprehensive sensitivity analyses.

**Results:**

Genetic liability to psoriasis showed no causal association with any of the following renal outcome: eGFR (β = -0.0005, *P* = 0.393), albuminuria (OR = 0.992, *P* = 0.819), CKD G3–G5 (OR = 1.012, *P* = 0.520), dialysis (OR = 0.940, *P* = 0.377), CKDi25 (OR = 1.013, *P* = 0.527), or Rapid3 (OR = 1.005, *P* = 0.728). No evidence of horizontal pleiotropy was detected.

**Discussion:**

Although prior observational studies have linked psoriasis to impaired kidney function, these associations are likely confounded by factors, such as nephrotoxic drug use (*e.g*., cyclosporine), metabolic comorbidities, or medical surveillance bias. In contrast, this MR study focused exclusively on genetic susceptibility to psoriasis, effectively avoiding such confounding factors. Our findings provide robust genetic evidence that psoriasis itself does not causally affect kidney function, as supported by the lack of significant associations across multiple renal endpoints. Moreover, genetic overlap between psoriasis and kidney disease risk appears minimal, reinforcing the notion that psoriasis is not an independent driver of renal damage. Clinically, screening should emphasize traditional risk factors, such as hypertension and diabetes. In psoriasis patients without these risk factors, frequent long-term kidney monitoring lacks a genetic rationale, which could optimize resource utilization and prevent unnecessary interventions.

**Conclusion:**

This MR study found no genetic causality between psoriasis and renal impairment across three domains: static kidney function (eGFR, albuminuria), end-stage damage (CKD G3–G5, dialysis), and dynamic progression (CKDi25, Rapid3). These findings challenge prior observational associations and highlight the importance of considering psoriasis-independent pathways in renal risk stratification.

## 1 INTRODUCTION

Psoriasis is a chronic, relapsing systemic condition characterized by inflammation [1], affecting an estimated 2–4% of the global population and driven primarily by immune- mediated processes [2, 3]. Although its precise pathogenesis is not fully understood, studies suggest that the onset or exacerbation of psoriasis results from an interplay of genetic, environmental, and immune factors [4-6]. More than 80 genetic loci, including PSORS1, HLA-Cw6, PSORS2, chromosome 6p21, and chromosome 17q, have been associated with psoriasis susceptibility [7]. Environmental factors, such as infections, stress, smoking, obesity, and alcohol consumption, also play a significant role in triggering or worsening the disease [8, 9].

Chronic kidney disease (CKD) affects approximately 11–13% of the global population, with stages G3–G5 substantially increasing the risk of mortality [10, 11]. Between 1990 and 2017, CKD-related deaths rose by 41.5%, exceeding 1.2 million fatalities in 2017 alone [12], highlighting its considerable burden on public health [13]. Given the incurable nature of CKD, current research emphasizes early detection of biomarkers to prevent the progression from mild or moderate CKD to end-stage renal disease [14, 15].

A growing body of evidence links psoriasis to multiple comorbidities, including metabolic syndrome, cardiovascular disease, and diabetes [16-18]. However, the relationship between psoriasis and renal impairment remains unclear. The term “psoriatic nephropathy” was first introduced in 2005 in case reports describing glomerulonephritis among psoriasis patients [19]. In the same year, population-based data indicated that, among 753 individuals with psoriasis, rates of diabetes and cardiovascular disease were elevated, whereas kidney failure occurred at levels consistent with national statistics [20]. Subsequent studies have similarly failed to establish a robust association between psoriasis and kidney disorders [20-22].

Mendelian randomization (MR) uses genetic variants as instrumental variables to reduce confounding and mitigate reverse causality, thereby providing stronger evidence for causal inference [23, 24]. Recent MR studies have examined psoriasis’s influence on other systemic diseases. For example, Shang *et al*. (2022) found that psoriasis may accelerate the progression of Parkinson’s disease and increase the risk of dementia and depression in affected individuals [25]. These findings suggest that psoriasis can impact multiple organ systems through systemic inflammation and immune dysregulation.

In this study, we applied MR methods leveraging large-scale genome-wide association study (GWAS) data and multiple statistical approaches to elucidate the causal relationship between psoriasis and dynamic changes in renal function, offering novel insights into clinical management and risk stratification.

## 2 MATERIALS AND METHODS

### 2.1 Psoriasis Data Source

The GWAS data for psoriasis used in this study were obtained from the FinnGen database (https://r9.finngen.fi/). FinnGen is an extensive genomic repository developed in Finland, established to explore potential associations between genetic variation and disease susceptibility among individuals of Finnish origin. All psoriasis cases and control subjects enrolled in this study were of European descent and had provided written consent. The dataset included 9,267 psoriasis cases and 364,071 control individuals. Psoriasis diagnoses were based on Finnish national health data, including clinical diagnostic criteria and patients' self-reported information. All data used in this study were derived from public databases. Therefore, this study did not set additional inclusion and exclusion criteria for the study subjects.

### 2.2 Sources of Data on Renal Function and CKD

The estimated glomerular filtration rate (eGFR) data were primarily obtained from the CKDGen Consortium, encompassing 765,348 individuals, predominantly of European ancestry [26]. For adults (>18 years), eGFR was calculated using the CKD-EPI formula, whereas for individuals under 18, the Schwartz formula was applied [27, 28], ensuring accurate and consistent eGFR measurements across age groups.

Albuminuria data were derived from the urinary albumin-to-creatinine ratio (UACR), with sex-specific thresholds defined as >17 mg/g (1.92 mg/mmol) for males and >25 mg/g (2.83 mg/mmol) for females. These thresholds were based on Warram *et al*., who established standards using the 95^th^ percentile of 218 healthy individuals to account for sex differences in creatinine excretion [29]. Although slightly lower than the KDIGO standard of ≥30 mg/g [30], this approach was adopted to reduce gender-related confounding. To further minimize potential confounding from diabetes, the albuminuria analysis included 46,061 non-diabetic participants of European ancestry [31].

Rapid progression to CKD (CKDi25) and rapid decline in renal function (Rapid3) were assessed using datasets from the CKDGen Consortium and a GWAS meta-analysis encompassing 42 studies, primarily involving European ancestry participants from the UK Biobank. Rapid3 is defined as an annual decline in eGFR exceeding 3 mL/min/1.73 m^2^, whereas CKDi25 represents a ≥25% decrease in eGFR from baseline, reflecting progression from no CKD to CKD. The Rapid3 dataset included 54,874 cases and 107,090 controls, while CKDi25 comprised 19,901 CKD patients and 175,244 controls [32]. Inclusion of these cohorts allowed a comprehensive assessment of psoriasis in relation to progressive renal function decline.

CKD stages G3–G5 (eGFR <60 mL/min/1.73 m^2^) data were derived from an integrative analysis of 23 European cohorts, including 41,395 CKD patients and 439,303 controls [26]. Dialysis data were obtained from the FinnGen dataset, comprising 1,004 dialysis patients and 363,177 European-ancestry controls. The CKD G3–G5 definition adhered to international standards, enabling evaluation of the association between severe renal impairment and psoriasis. A summary of all data sources is provided in Table **1**.

### 2.3 Selection of Genetic Instruments

The selection of genetic instruments involved several steps. First, SNPs significantly associated with the target trait were extracted from GWAS studies with a *p*-value threshold of 5×10^−8^. Second, these genetic instruments must have a linkage disequilibrium (LD) parameter (r^2^) less than 0.001 and be within 1 MB of the index variant [33]. This ensured that the selected variants were minimally correlated with other closely linked variants, representing independent genetic signals. Only variants available across all traits were used, and proxy replacements for missing variants were excluded. Before each MR analysis, the Mendelian Randomization Pleiotropy RESidual Sum and Outlier (MR-PRESSO) procedure was applied to detect and remove influential outliers [34]. Finally, F-statistics were computed using the equation: F = R^2^ (N-K-1)/[K(1-R^2^)], to confirm that the instrumental SNPs exhibited F-values exceeding the threshold of 10, thereby satisfying the requirement for a strong genetic instrument. The research framework is illustrated in Fig. (**1**).

### 2.4 Mendelian Randomization Analyses

In this study, the Mendelian Randomization (MR) framework was adopted, primarily utilizing the inverse-variance weighted (IVW) method within a random-effects model to estimate causal effects. To strengthen the robustness of these estimates, complementary approaches, such as the MR Egger and weighted median methods, were also employed, despite their comparatively lower statistical efficiency [35]. Assessment of heterogeneity was conducted using Rucker’s Q test for the MR Egger approach and Cochran’s Q test for the IVW method, with the latter serving as the primary metric. A *P*-value greater than 0.05 indicated no significant heterogeneity [36]. Horizontal pleiotropy was evaluated using MR Egger intercept analysis and leave-one-out analysis, and funnel plots were employed to detect potential directional pleiotropy. The impact of individual SNPs on causal relationships was examined through leave-one-out analysis, while outliers were identified and removed using MR-PRESSO's global test and distortion test, followed by a re-evaluation of the causal estimates. All analyses were performed in R (v4.1.2) using the TwoSampleMR and MR-PRESSO toolkits.

## 3 RESULTS

Following the selection process, 30 index SNPs were ultimately included for genetic prediction of psoriasis. All instrumental variants demonstrated strong associations (F-statistics >10). The outlier SNP rs13153019 showed overlap in CKD G3-G5 and eGFR analyses. Palindromic SNPs rs2664280 and rs7310615 with intermediate allele frequencies were excluded, with rs7310615 having been previously removed as an eGFR-associated variant (Table **2**). These selected SNPs proceeded to Mendelian randomization analysis (Supplementary Tables **S1-S6**).

Our comprehensive MR analysis evaluated genetically predicted psoriasis against six renal function parameters. Random-effects inverse variance weighted (IVW) estimates revealed no causal association between psoriasis and renal outcomes across static measures (eGFR: β=-0.0005, *P*=0.393, albuminuria: OR=0.992, *P*=0.819), end-stage renal disease (CKD G3-G5: OR=1.012, *P*=0.520; dialysis: OR=0.940, *P*=0.377), or progression markers (CKDi25: OR=1.013, *P*=0.527, Rapid3: OR=1.005, *P*=0.728) (Fig. **2**). Cochran's Q test identified heterogeneity in CKD G3-G5 and eGFR analyses (*P*<0.05) (Table **2**), while MR-Egger intercept tests showed no evidence of horizontal pleiotropy (all *P*>0.05) (Supplementary Table **S7**). Leave-one-out sensitivity analysis (Supplementary Fig. **S1**) and funnel plots (Supplementary Fig. **S2**) demonstrated no influential individual SNPs.

## 4 DISCUSSION

CKD is a progressive disorder characterized by a gradual loss of renal function, clinically classified into five stages based on glomerular filtration rate thresholds [37]. Stages G3-G5 represent moderate to severe disease with substantial functional deterioration [38]. Albuminuria serves as a critical biomarker of glomerular injury, with its severity correlating positively with risks of renal function decline [39]. eGFR, which quantifies the kidney's filtration capacity for waste products, provides essential prognostic information, as its reduction reflects progression of functional impairment [40]. Emerging dynamic parameters further characterize renal trajectory. Rapid3 (eGFR decline >3 mL/min/1.73 m^2^/year) and CKDi25 (≥25% eGFR reduction from baseline with progression to CKD) offer standardized metrics for monitoring disease evolution. These parameters collectively enable a comprehensive assessment of renal functional status and progression dynamics.

This MR study provides robust genetic evidence that psoriasis does not exert a causal effect on renal dysfunction, as demonstrated by null associations across multiple endpoints, including eGFR decline, albuminuria, CKD G3–G5 progression, and dialysis risk. Our findings contrast with prior observational studies reporting increased kidney disease prevalence in psoriasis patients. A landmark cohort study by Gelfand *et al*. identified a 4.4-fold elevated mortality risk from kidney disease in severe psoriasis, while Yang *et al*. observed 59% higher renal failure prevalence in severe cases compared to controls [8, 16]. However, these observational designs likely conflated psoriasis severity with confounders, such as nephrotoxic therapies (*e.g*., cyclosporine), metabolic comorbidities (*e.g*., diabetes), or healthcare surveillance bias. For instance, systemic inflammation in psoriasis correlates strongly with obesity and hypertension, which are established drivers of CKD. However, our MR analysis, which isolates genetic liability from environmental factors, suggests no intrinsic renal toxicity attributable to psoriasis pathophysiology. This aligns with recent genetic studies showing minimal overlap between psoriasis-associated loci (*e.g*., IL23R, TNFAIP3) and kidney disease risk variants [41].

The discordance between observational and MR findings may reflect the mechanistic decoupling of psoriasis-specific inflammation from direct renal injury pathways. While the IL-17/IL-23 axis activation drives psoriatic plaque formation [42], animal models reveal that isolated IL-17 overexpression induces minimal glomerular damage unless synergized with angiotensin II or a high-salt diet [43]. Furthermore, psoriasis-associated cytokines like TNF-α exhibit dual roles: though pro-inflammatory in the skin, they may paradoxically protect the renal endothelium by suppressing vascular adhesion molecules [44, 45]. Such tissue-specific effects could explain why genetic predisposition to psoriasis does not translate into kidney dysfunction.

Methodologically, our study addresses key limitations of prior research. Earlier cross-sectional analyses lacked the power to distinguish transient proteinuria from progressive CKD and were susceptible to reverse causation. By contrast, MR leverages lifelong genetic exposure to psoriasis, minimizing confounding by disease duration or treatment effects. The consistency of null results across renal endpoints (static/dynamic measures, mild/severe outcomes) and sensitivity analyses (Cochran’s Q *P* > 0.05 for most traits) reinforces the robustness.

## 5 LIMITATIONS

Nevertheless, three limitations warrant consideration: First, European-biased GWAS data may limit generalizability to populations with distinct genetic-environmental interactions. Second, eGFR and albuminuria capture global renal function but miss localized tubular injury potentially linked to subclinical inflammation. Third, while MR examines lifelong risk, it cannot exclude transient renal effects during psoriasis flares.

Clinically, these findings argue against routine nephrological screening in psoriasis patients without traditional risk factors. However, vigilance remains critical in subgroups receiving nephrotoxic therapies or with severe metabolic syndrome. Future research should prioritize: 1) Study of the association between different clinical subtypes of psoriasis and renal function impairment. 2) Drug-specific MR analyses to disentangle medication effects from disease biology. 3) Exploration of non-linear relationships where extreme psoriasis severity thresholds may overwhelm compensatory mechanisms. Finally, given the considerable annual cost of laboratory monitoring for psoriasis patients, reducing unnecessary routine tests is expected to significantly reduce medical expenditures. We suggest reducing the frequency of renal function monitoring to once every 1-2 years for such low-risk patients, which has notable economic value.

## CONCLUSION

This MR study revealed no genetic causality between psoriasis and renal impairment across three domains: static function (eGFR, albuminuria), end-stage damage (CKD G3-G5, dialysis), or dynamic progression (CKDi25, Rapid3). These findings challenge observational associations and highlight the need to reconsider psoriasis-independent pathways in renal risk stratification.

## Figures and Tables

**Fig. (1):**
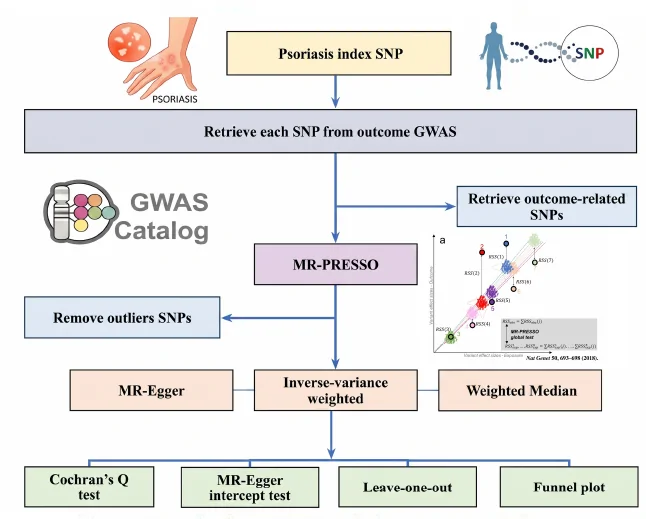
Technology roadmap. **Abbreviations:** SNP, single-nucleotide polymorphism. GWAS, genome-wide association study. MR-PRESSO, Mendelian randomization pleiotropy RESidual sum and outlier.

**Fig. (2):**
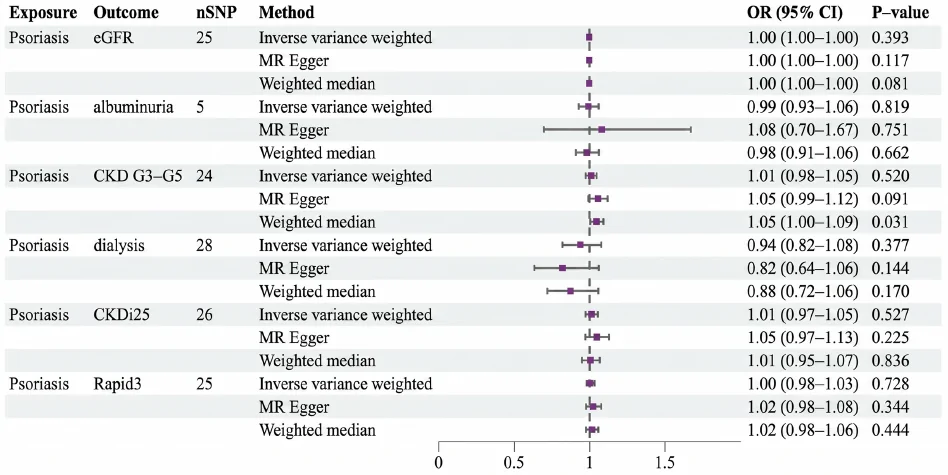
Forest plot showing causal associations between genetically predicted psoriasis and six kidney-related phenotypes. The Bonferroni-corrected significance threshold was set at *P* < 0.0083. **Abbreviations:** SNP, single-nucleotide polymorphism. OR, odds ratio. CI, confidence interval.

**Table 1:** Data sources.

**Traits**	**Data Sources**	**Sample Size (Cases/Controls)**	**Ancestry**	**Gender**
**Exposure**	-	-	-	-
Psoriasis	FinnGen	9,267 / 364,071	European	Men and women
**Outcomes**	-	-	-	-
eGFR	CKDGen Consortium	765,348	European	Men and women
Albuminuria	CKDGen Consortium	5,828 / 46,061	European	Men and women
CKD G3-G5	CKDGen Consortium	41,395 / 439,303	European	Men and women
Dialysis	FinnGen	1,004 / 363,177	European	Men and women
CKDi25	CKDGen Consortium	19,901 / 175,244	European	Men and women
Rapid3	CKDGen Consortium	54,874 / 107,090	European	Men and women

**Table 2:** Summary of instrumental variable characteristics and sensitivity test.

**Direction**		-	-	-	**Heterogeneity Test**		**Pleiotropy Test**
**Exposure**	**Outcome**	Outcome-related SNPs	Palindromic SNP	Distortion Test (Outliers)	Cochran's Q Test (IVW)	Rucker's Q Test (MR-Egger)	Egger Intercept (*P*-value)
**Psoriasis**	**eGFR**	1	1	1	0.026	0.040	0.182
	**Albuminuria**	0	1	0	0.288	0.190	0.725
	**CKD G3-G5**	0	2	1	0.029	0.059	0.105
	**Dialysis**	0	2	0	0.191	0.214	0.234
	**CKDi25**	0	2	0	0.93	0.939	0.294
	**Rapid3**	0	2	0	0.457	0.449	0.360
**Psoriasis**	-	-	-	-	-	-	-
	**eGFR**	rs7310615	rs2664280	rs13153019	-	-	-
	-	-	-	-	-	-	-
	**Albuminuria**	-	rs2664280	-	-	-	-
		-		-	-	-	-
	-	-	-	-	-	-	-
	**CKD G3-G5**	-	rs2664280	-	-	-	-
		-	rs7310615	rs13153019	-	-	-
	-	-	-	-	-	-	-
	**Dialysis**	-	rs2664280	-	-	-	-
		-	rs7310615	-	-	-	-
	-	-	-	-	-	-	-
	**CKDi25**	-	rs2664280	-	-	-	-
		-	rs7310615	-	-	-	-
	-	-	-	-	-	-	-
	**Rapid3**	-	rs2664280	-	-	-	-
		-	rs7310615	-	-	-	-

## Data Availability

All data generated or analyzed during this study are included in this published article.

## LIST OF ABBREVIATIONS

MR: Mendelian Randomization
eGFR: Estimated Glomerular Filtration Rate
CKD: Chronic Kidney Disease
CKDi25: ≥25% decline in eGFR with progression from no CKD to CKD
IVW: Inverse-variance Weighted
SNP: Single-Nucleotide Polymorphism

## STANDARDS OF REPORTING

STROBE guidelines were followed.
