# Supplementary material for: Mendelian Randomization Analysis of Psoriasis and Multiparameter Renal Function Trajectories
Source: Endocr Metab Immune Disord Drug Targets. 2026 May 21;26:E18715303426134. doi: 10.2174/0118715303426134251202143308 (PMC13670195; doi:10.2174/0118715303426134251202143308)
Supplement: Supplementary file 1 [file EMIDDT-26-E18715303426134_SD1.pdf]

Supplementary Material

Mendelian Randomization Analysis of Psoriasis and Multiparameter Renal Function Trajectories

Zhihao Huang[1] and Shengxiu Liu[1,*]

[1]*Department of Dermatology, Anhui Medical University, Hefei, Anhui, 230022, China*

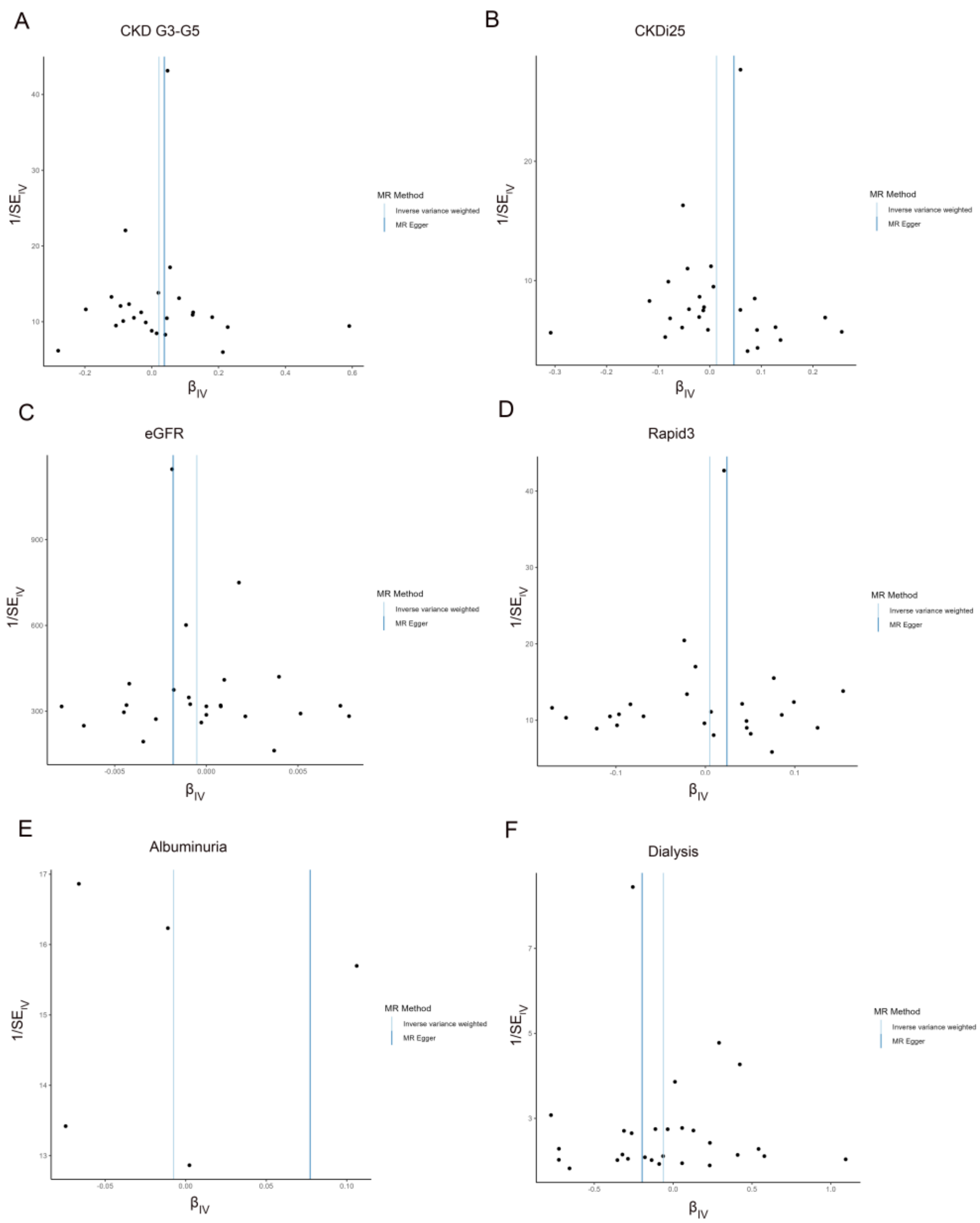

Supplementary Fig S1: Leave-one-out sensitivity analysis. Genetically predicted psoriasis on (**A**) CKD G3-G5, (**B**) CKDi25, (**C**) eGFR, (**D**) Rapid3, (**E**) Albuminuria, (**F**) Dialysis.

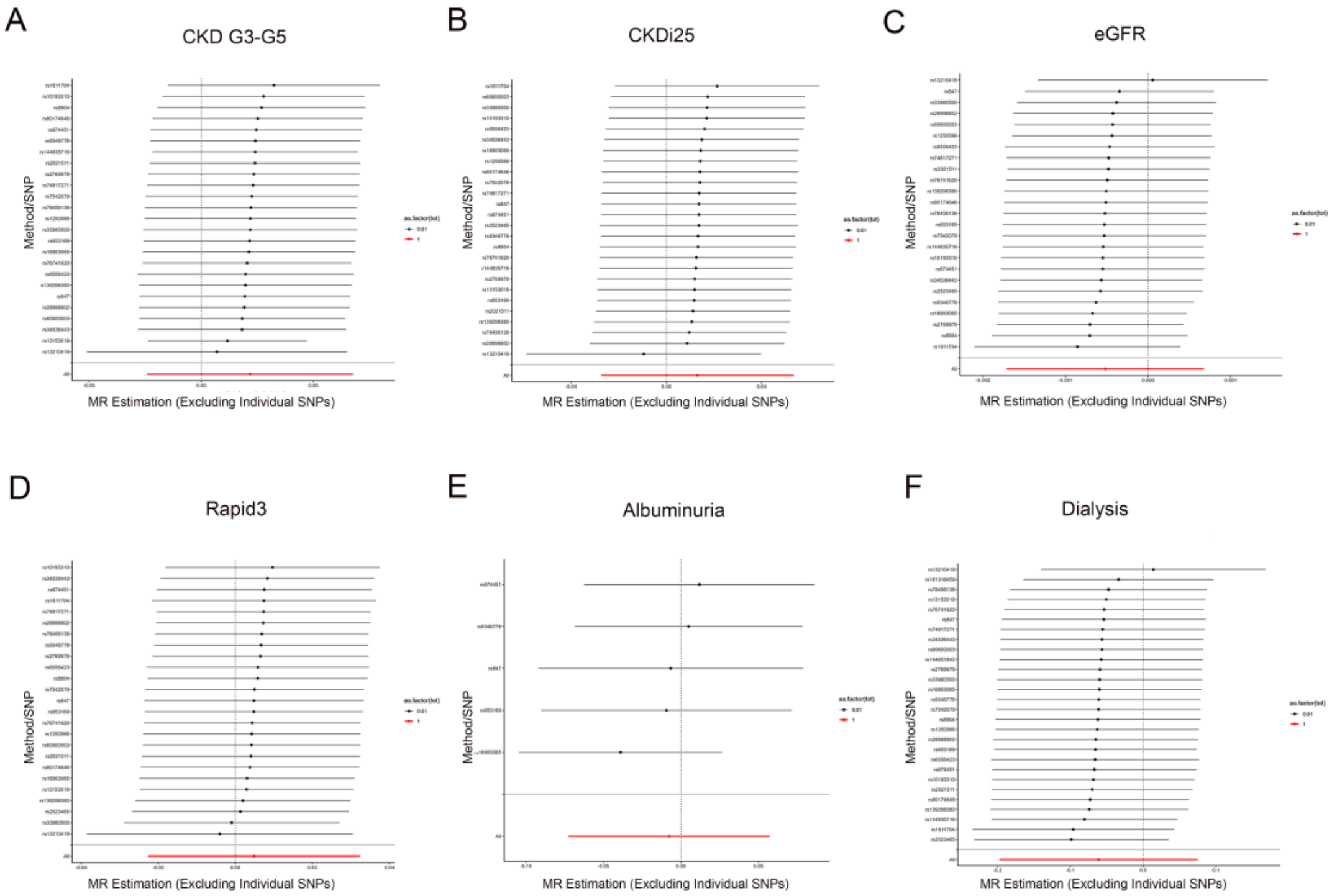

Supplementary Fig S2: Funnel plot of psoriasis and six renal function phenotypes. Genetically predicted psoriasis on (**A**) CKD G3-G5, (**B**) CKDi25, (**C**) eGFR, (**D**) Rapid3, (**E**) Albuminuria, (**F**) Dialysis.

Table S1. Mendelian randomization analysis of Psoriasis and CKD G3-G5.

SNP	effect_allele.	other_allele.	beta.Psoriasis	beta.CKD	se.CKD	pval.CKD	pval.Psoriasis
rs10193310	A	G	0.126764	-0.025	0.0109	0.02161	9.34E-14
rs1250566	A	G	-0.088896	-0.0013	0.0105	0.8997	6.64E-09
rs13210419	A	G	0.802509	0.038	0.0186	0.04137	2.61E-228
rs139298380	A	G	0.313294	0.0256	0.0239	0.2833	4.59E-14
rs144835716	T	A	0.243289	-0.0681	0.0394	0.08396	4.48E-11
rs1611704	T	C	0.224967	-0.0177	0.0102	0.08247	3.43E-49
rs16903065	A	C	-0.141275	-0.0064	0.0135	0.6353	5.01E-09
rs2021511	T	C	-0.108961	0.0093	0.0108	0.3916	2.5E-10
rs2769979	C	T	-0.0957142	0.0051	0.0091	0.5723	2.64E-10
rs28998802	A	G	0.160654	0.0199	0.0143	0.1631	7.83E-18
rs33980500	T	C	0.237708	0.0048	0.0172	0.7808	8.26E-19

rs34536443	C	G	-0.281948	-0.051	0.0266	0.05534	2.73E-09
rs60600003	G	T	0.149572	0.034	0.0161	0.03439	2.62E-10
rs653169	G	A	-0.0861892	-0.0035	0.0104	0.736	1.14E-08
rs6556423	T	C	-0.180408	-0.0099	0.0105	0.3457	3.05E-32
rs674451	C	T	0.128161	-0.0087	0.0104	0.4016	1.29E-16
rs74817271	A	G	0.224803	-0.0071	0.02	0.7233	4.12E-17
rs7542079	C	T	0.0950954	-0.0017	0.0096	0.8592	2.87E-10
rs76741620	G	A	0.174314	0.0371	0.0291	0.2025	1.06E-08
rs78456138	T	C	-0.364637	0	0.0414	0.9996	6.61E-10
rs80174646	T	G	-0.227133	0.0212	0.0188	0.2575	2.81E-09
rs847	C	T	0.126621	0.0155	0.0116	0.1794	8.63E-16
rs8904	A	G	-0.126143	0.0152	0.0095	0.1092	2.83E-16
rs9346778	T	C	-0.116749	0.0125	0.0123	0.3063	1.55E-08

Table S2. Mendelian randomization analysis of Psoriasis and eGFR.

SNP	effect_allele.	other_allele.	beta.psoriasis	beta.eGFR	se.eGFR	pval.eGFR	pval.psoriasis
rs10193310	A	G	0.126764	0.0001	0.0004	0.7387	9.34E-14
rs1250566	A	G	-0.088896	0.0004	0.0003	0.1915	6.64E-09
rs13210419	A	G	0.802509	-0.0015	0.0007	0.02903	2.61E-228
rs139298380	A	G	0.313294	-0.0003	0.0009	0.7721	4.59E-14
rs144835716	T	A	0.243289	0.0009	0.0015	0.5423	4.48E-11
rs1611704	T	C	0.224967	0.0004	0.0003	0.2912	3.43E-49
rs16903065	A	C	-0.141275	-0.0011	0.0005	0.02334	5.01E-09
rs2021511	T	C	-0.108961	0.0003	0.0004	0.4937	2.5E-10
rs2523465	C	G	0.204877	0.0002	0.0005	0.6636	1.55E-39
rs2769979	C	T	-0.0957142	-0.0007	0.0003	0.03883	2.64E-10
rs28998802	A	G	0.160654	-0.0007	0.0005	0.1489	7.83E-18
rs33980500	T	C	0.237708	-0.001	0.0006	0.1197	8.26E-19
rs34536443	C	G	-0.281948	-0.0006	0.001	0.5249	2.73E-09

rs60600003	G	T	0.149572	-0.001	0.0006	0.08907	2.62E-10
rs653169	G	A	-0.0861892	0	0.0003	0.9498	1.14E-08
rs6556423	T	C	-0.180408	0.0002	0.0003	0.6277	3.05E-32
rs674451	C	T	0.128161	0.0001	0.0004	0.8566	1.29E-16
rs74817271	A	G	0.224803	-0.0004	0.0006	0.5498	4.12E-17
rs7542079	C	T	0.0950954	0	0.0003	0.9483	2.87E-10
rs76741620	G	A	0.174314	-0.0006	0.0009	0.5069	1.06E-08
rs78456138	T	C	-0.364637	0.0001	0.0014	0.916	6.61E-10
rs80174646	T	G	-0.227133	0.0002	0.0007	0.8027	2.81E-09
rs847	C	T	0.126621	-0.001	0.0004	0.008883	8.63E-16
rs8904	A	G	-0.126143	-0.0005	0.0003	0.0861	2.83E-16
rs9346778	T	C	-0.116749	-0.0006	0.0004	0.1546	1.55E-08

Table S3. Mendelian randomization analysis of Psoriasis and Albuminuria.

SNP	effect_allele.	other_allele.	beta.psoriasis	beta.Albuminuria	se.Albuminuria	pval.Albuminuria	pval.psoriasis
rs16903065	A	C	-0.141275	-0.015	0.009	0.099	5.01E-09
rs653169	G	A	-0.0861892	-0.0002	0.0067	0.97	1.14E-08
rs674451	C	T	0.128161	-0.0085	0.0076	0.27	1.29E-16
rs847	C	T	0.126621	-0.0014	0.0078	0.86	8.63E-16
rs9346778	T	C	-0.116749	0.0087	0.0087	0.32	1.55E-08

Table S4. Mendelian randomization analysis of Psoriasis and dialysis.

SNP	effect_allele.	other_allele.	beta.psoriasis	beta.dialysis	pval.dialysis	se.dialysis	pval.psoriasis
rs10193310	A	G	0.126764	0.0295803	0.571771	0.0523132	9.34E-14
rs1250566	A	G	-0.088896	-0.00507418	0.911652	0.045732	6.64E-09
rs13153019	C	T	0.101863	-0.0737901	0.142584	0.0503261	6.67E-10
rs13210419	A	G	0.802509	-0.205028	0.0309272	0.0950082	2.61E-228
rs139298380	A	G	0.313294	0.169711	0.216921	0.137444	4.59E-14

rs144651842	A	G	0.171788	-0.0491925	0.557596	0.0838867	1.58E-10
rs144835716	T	A	0.243289	0.266218	0.0259251	0.119523	4.48E-11
rs1611704	T	C	0.224967	0.0655275	0.16396	0.0470786	3.43E-49
rs16903065	A	C	-0.141275	0.0192535	0.783394	0.0700383	5.01E-09
rs181316459	C	G	0.330442	-0.255868	0.0172088	0.107408	3.99E-24
rs2021511	T	C	-0.108961	-0.0445812	0.381437	0.0509353	2.5E-10
rs2523465	C	G	0.204877	0.0866423	0.0709545	0.0479808	1.55E-39
rs2769979	C	T	-0.0957142	0.0171149	0.709421	0.0459293	2.64E-10
rs28998802	A	G	0.160654	0.00918505	0.874028	0.0579338	7.83E-18
rs33980500	T	C	0.237708	-0.0266911	0.757672	0.0865079	8.26E-19
rs34536443	C	G	-0.281948	0.0906076	0.490025	0.131264	2.73E-09
rs60600003	G	T	0.149572	-0.0528454	0.475986	0.0741405	2.62E-10
rs653169	G	A	-0.0861892	-0.0200042	0.660564	0.0455537	1.14E-08
rs6556423	T	C	-0.180408	-0.00212561	0.96372	0.0467305	3.05E-32
rs674451	C	T	0.128161	0.0165033	0.726765	0.047229	1.29E-16
rs74817271	A	G	0.224803	-0.0591081	0.485844	0.0848116	4.12E-17
rs7542079	C	T	0.0950954	-0.00604705	0.893238	0.0450574	2.87E-10
rs76741620	G	A	0.174314	-0.114474	0.231445	0.0956625	1.06E-08
rs78456138	T	C	-0.364637	0.263991	0.0982291	0.159656	6.61E-10
rs80174646	T	G	-0.227133	-0.131396	0.222152	0.107629	2.81E-09
rs847	C	T	0.126621	-0.0394393	0.399241	0.0467857	8.63E-16
rs8904	A	G	-0.126143	0.00434782	0.924641	0.0459651	2.83E-16
rs9346778	T	C	-0.116749	0.0103057	0.865	0.060617	1.55E-08

Table S5. Mendelian randomization analysis of Psoriasis and CKDI25.

SNP	effect_allele.	other_allele.	beta.psoriasis	beta.CDKI25	se.CDKI25	pval.CDKI25	pval.psoriasis
rs10193310	A	G	0.126764	-0.014809116	0.0153	0.3341	9.34E-14
rs1250566	A	G	-0.088896	0.007670506	0.0169	0.6495	6.64E-09
rs13153019	C	T	0.101863	0.009343515	0.0174	0.5915	6.67E-10
rs13210419	A	G	0.802509	0.047837329	0.029	0.1005	2.61E-228

rs139298380	A	G	0.313294	0.027323296	0.0369	0.4594	4.59E-14
rs144835716	T	A	0.243289	0.022552404	0.0559	0.6855	4.48E-11
rs1611704	T	C	0.224967	-0.011667805	0.0138	0.3991	3.43E-49
rs16903065	A	C	-0.141275	0.005683816	0.0186	0.7581	5.01E-09
rs2021511	T	C	-0.108961	-0.013896105	0.0179	0.4394	2.5E-10
rs2523465	C	G	0.204877	0.000499875	0.0183	0.98	1.55E-39
rs2769979	C	T	-0.0957142	-0.005683816	0.0127	0.657	2.64E-10
rs28998802	A	G	0.160654	0.035946127	0.0233	0.1235	7.83E-18
rs33980500	T	C	0.237708	-0.019080888	0.024	0.4249	8.26E-19
rs34536443	C	G	-0.281948	0.02166364	0.0413	0.5999	2.73E-09
rs60600003	G	T	0.149572	-0.046119936	0.0266	0.08235	2.62E-10
rs653169	G	A	-0.0861892	-0.011829752	0.0172	0.4926	1.14E-08
rs6556423	T	C	-0.180408	0.007769737	0.0164	0.6359	3.05E-32
rs674451	C	T	0.128161	-0.001598721	0.0171	0.9237	1.29E-16
rs74817271	A	G	0.224803	-0.004610613	0.0324	0.8871	4.12E-17
rs7542079	C	T	0.0950954	-0.005087039	0.0157	0.7437	2.87E-10
rs76741620	G	A	0.174314	0.012781334	0.0427	0.7647	1.06E-08
rs78456138	T	C	-0.364637	-0.093322157	0.064	0.1453	6.61E-10
rs80174646	T	G	-0.227133	0.004489905	0.0263	0.8656	2.81E-09
rs847	C	T	0.126621	-0.001399021	0.0163	0.9294	8.63E-16
rs8904	A	G	-0.126143	-0.000900405	0.0133	0.9445	2.83E-16
rs9346778	T	C	-0.116749	0.00039992	0.0199	0.9824	1.55E-08

Table S6. Mendelian randomization analysis of Psoriasis and Rapid3.

SNP	effect_allele.	other_allele.	beta.psoriasis	beta.Rapid3	se.Rapid3	pval.Rapid3	pval.psoriasis
rs10193310	A	G	0.126764	-0.021734492	0.0109	0.04742	9.34E-14
rs1250566	A	G	-0.088896	-0.004510155	0.0108	0.6765	6.64E-09
rs13153019	C	T	0.101863	0.012781334	0.0113	0.2579	6.67E-10

rs13210419	A	G	0.802509	0.016758784	0.0188	0.3713	2.61E-228
rs139298380	A	G	0.313294	0.031014054	0.0253	0.2197	4.59E-14
rs1611704	T	C	0.224967	-0.005314095	0.011	0.6318	3.43E-49
rs16903065	A	C	-0.141275	-0.012072581	0.0132	0.3602	5.01E-09
rs2021511	T	C	-0.108961	-0.005012542	0.011	0.6503	2.5E-10
rs2523465	C	G	0.204877	0.015676479	0.0132	0.2366	1.55E-39
rs2769979	C	T	-0.0957142	0.006621876	0.0091	0.4653	2.64E-10
rs28998802	A	G	0.160654	-0.015519812	0.0149	0.2986	7.83E-18
rs33980500	T	C	0.237708	0.036621183	0.0172	0.03342	8.26E-19
rs34536443	C	G	-0.281948	0.043921187	0.0273	0.1079	2.73E-09
rs60600003	G	T	0.149572	0.006923915	0.0166	0.6757	2.62E-10
rs653169	G	A	-0.0861892	-0.00079968	0.0107	0.9397	1.14E-08
rs6556423	T	C	-0.180408	0.001998003	0.0106	0.8484	3.05E-32
rs674451	C	T	0.128161	-0.010742097	0.0106	0.3152	1.29E-16
rs74817271	A	G	0.224803	-0.023985363	0.0214	0.2608	4.12E-17
rs7542079	C	T	0.0950954	-0.0001	0.0099	0.9912	2.87E-10
rs76741620	G	A	0.174314	0.012983928	0.0298	0.6622	1.06E-08
rs78456138	T	C	-0.364637	0.044303926	0.0409	0.2788	6.61E-10
rs80174646	T	G	-0.227133	-0.009343515	0.0187	0.6215	2.81E-09
rs847	C	T	0.126621	0.00080032	0.0114	0.9448	8.63E-16
rs8904	A	G	-0.126143	0.002596626	0.0094	0.7827	2.83E-16
rs9346778	T	C	-0.116749	0.011533236	0.0125	0.3594	1.55E-08

Supplementary Table S7: Pleiotropy (Egger-Intercept) and Heterogeneity (Q-Statistic) Test.

	Pleiotropy Test			Heterogeneity Test		
**Outcome**	**Egger-Intercept**	**S.E.**	**P**	**Q**	**Q-df**	**Q-P**
**eGFR**	0.0003	0.0002	0.182	39.136	24	0.0264
**Albuminuria**	-0.0102	0.0263	0.725	4.996	4	0.2876
**CKD G3-G5**	-0.0096	0.0057	0.105	37.485	23	0.0289
**Dialysis**	0.0288	0.0236	0.2338	33.193	27	0.1907
**CKDi25**	-0.0075	0.007	0.294	15.458	25	0.930
**Rapid3**	-0.0043	0.0046	0.360	24.073	24	0.457
